# General dental practitioners' perceptions of shared decision making: a qualitative study

**DOI:** 10.1038/s41415-022-3980-9

**Published:** 2022-02-25

**Authors:** Natasha Hayer, Hoda S. Wassif

**Affiliations:** 4141591204001 Dental Surgery, 180 Northridge Way, Hemel Hempstead, HP1 2AL, UK; 4141591204002 University of Bedfordshire, Healthcare Practice, Putteridge Bury Campus, Hitchin Road, Luton, LU2 8LE, UK

## Abstract

**Introduction** As patient-centred care becomes more of the focus in healthcare, informed consent is receiving more attention in dental practice as the pinnacle principle in patient care. Shared decision making or person-centred care appears to be taking a back foot in dentistry.

**Aim** This study aims to gain insight into the current views and perceptions of shared decision making by general dental practitioners and how it can be utilised in daily practice.

**Method** Empirical qualitative data were collected using semi-structured interviews of nine dentists working in general practice, with an average of 30 minutes for each interview. The data were analysed using thematic analysis.

**Results** Overall, there was a misunderstanding of what constitutes shared decision making among dental practitioners, with communication focused more on information provision rather than collaborative discussion. There were barriers which prevented full discussions with patients from occurring, some of which directly conflicted with the focus of shared decision making.

**Conclusion** There is a need to provide more clarity of what shared decision making is and how it can facilitate person-centred care in dental practice. Dental practitioners felt that standards and guidelines were not clear; therefore, they were confused as to what was expected of them with regards to communication. More support, in the form of standardised decision-making aids, is needed to assist dental practitioners to streamline the delivery of shared decision making in primary care.

## Key points

- Explores the subject of shared decision making in dental practice.
- Presents an insight into the current views and perceptions of shared decision making by general dental practitioners.
- Discusses some of the barriers to using shared decision making in dental practice with some suggestions about how to better support dental practitioners.

## Introduction

Paternalistic care has long dominated medical care since the very conception of medicine. The key feature of paternalistic care is that of 'doctor knows best',^1^ with patients having mostly a passive role in the decision-making process and relying heavily on implicit trust that the doctor will always act in their best interest. The view that the doctor is bound by a specific code of ethics only reinforces this trust. It is also generally viewed that because the doctor has the most knowledge regarding all things medical, that they are best placed to solely devise a patient's medical care.^2^

Shared decision making (SDM) involves the clinician actively seeking information regarding the patient in a social and psychological context. In return, the patient shares their values, beliefs, knowledge about their disease and preferences.^3^ The goal is to select treatment that is appropriate for the patient based on this interactive approach. Research^2^^,^^3^ outlined that for a discussion to be truly shared, four features must be employed: 'both the clinician and patient are involved in all processes; both parties share information; both parties express treatment preferences; and agreement is reached'.^4^ Therefore, SDM evolves past informed consent, allowing clinicians and patients to 'think, talk and feel through the situation',^5^ thus providing a prologue and epilogue to the patients' treatment 'story'. Its importance can only be explained by overwhelming evidence from extensive research, emphasising better communication having a direct link to better patient healthcare outcomes.^6^^,^^7^^,^^8^^,^^9^

The term is widely researched and developed in medicine, though not so much in dentistry. Given that there is a strong UK government backing^9^^,^^10^^,^^11^ for driving medicine toward the patient- or person-centred care (PCC) model, there seems to be a delay for dentistry to follow suit. With informed consent being the pinnacle principle in dental ethics, PCC appears to be taking a back foot in dentistry. Tremendous research has been done regarding the concept and implementation of SDM by general medical practitioners, resulting in continued improvements to aid its development. However, there is a noticeable deficit in research with regards to the same for general dental practitioners (GDPs).

Interestingly, although it can be described, SDM has no universally accepted definition in healthcare, nor can it be measured.^12^ Both these factors make it a difficult concept to implement,^13^ creating a gap between what patients expect from their healthcare experience versus what and how this experience can be delivered by their practitioner.

Much of the literature defines informed consent as a piece of a wider framework that is SDM.^14^ The axiom 'informed consent' has multiple interpretations with no clear description as to what it entails. Dental Protection^15^ emphasises the need to focus on gaining consent based on ensuring patient understanding rather than providing information alone. NHS England appears to suggest an approach that relies on information provision as well as the need for patient preference and treatment options to align, provided they are 'clinically valid'.^16^ The General Dental Council (GDC) outlined within its guidance^17^ the need for clear documentation of discussions, plus signatures confirming decisions agreed with patients, but the guidance as to how remains open for debate. The goal of informed consent is to gain a 'yes' or 'no' answer based on a discussion of pros and cons and a list of treatment options, some of which may or may not be relevant to the patient.^18^ Therefore, informed consent is the legal process required regardless of choice and SDM is the process which facilitates the choice process.^19^

In dentistry, the term SDM is an emerging one, with informed consent dominating the decision making aspect of care. Indeed, informed consent has become perhaps more synonymous with avoiding litigation and the consent communication process has been unsuccessfully implemented at times.^20^ There is very little in the dental literature describing the true importance of assisting the patient towards a decision that promotes their health in a more holistic scope. What appears to be missing from the literature is the notion that perhaps the dentist employing the principles of SDM fully may ease the practice of defensive dentistry. There is some evidence that SDM may result in lower litigation rates.^21^

The aim of this study was to ascertain the views of GDPs regarding SDM and understanding the potential influence of SDM on enhanced patient care and how it can be implemented in general dental practice.

## Methods

As the aim for this study was to obtain practitioners' views and perceptions about SDM, a qualitative approach (interpretive paradigm)^22^^,^^23^ was adopted. As we set out to explore ideas, perceptions and depth of views, a qualitative rather than a quantitative approach was needed and data later analysed using thematic analysis.^24^^,^^25^^,^^26^ Ethical approval was obtained from the University of Bedfordshire. Data were collected using semi-structured interviews^27^ which were used to produce rich and deep data to answer the research question and explore the different facets influencing clinicians' perceptions and attitudes. Semi-structured interviews were also chosen as they allowed the flexibility in exploring the topic in more depth with participants, unlike questionnaires or structured interviews.

Purposive sampling^28^ was adopted and recruitment was carried out via the research participation invitation displayed on a private dental online forum on Facebook. Participation in the study was voluntary and no incentives were offered to participants for taking part. An information sheet explaining the outline of the study together with a consent form were shared with the participants. Semi-structured interviews were carried out via Skype allowing flexibility for participants with data saturation reached after nine interviews. Interviews ranged between 25-45 minutes each with an average of 30 minutes, with a total of approximately 4 hours of collected data. Participants were all dentists who had experience of working at least one year post dental foundation training. Each participant was designated P (1-x). Interview questions^22^ were formulated to guide the interview, rather than to lead it rigidly.

## Results

Nine dentists (of which six were men and three were women) took part in the study, with eight participants working in mixed practices, offering both private and NHS treatment and one participant offering 100% private dental treatment. Experience levels ranged from 3-16 years and participants predominantly qualified from the UK, apart from one participant who originally graduated from India. Semi-structured interviews were conducted, recorded, transcribed and coded by the first author. Transcribing the interviews took place within the first 48 hours following the interview which permitted immersion into the raw data, allowing observations of emerging patterns with data analysed using thematic analysis.^24^^,^^25^^,^^26^

As participants considered their perception and understanding of SDM in dental practice, three overarching themes were derived: communication with patients, perception of regulatory bodies and dental care structure.

### Communication with patients

The data supported an awareness from the participants of receptiveness to conversation among their patients. This played an important role in how discussions tended to evolve. For instance, conversation that involved 'small talk' or talk that was non-clinical allowed rapport building to begin, which, in turn, resulted in how much information was given to the patient:

- *'[…] you almost taper down or taper up the amount of information that [patients] need or the chattiness that they need […] some patients just aren't chatty are they? Some patients just see it as a part of their day and that's fine, but some it's actually a fairly big thing'* P6.

Most participants who took part indicated that they tended to give all the options, risks and benefits to the patient and few tailored their options, risks and benefits to the patient based on interaction:

- *'We'll go through the option of no treatment versus treatment and what happens if there's no treatments. Then we will go through all the options regardless of private or NHS. We'll go through the cosmetic side of it and an option to see a specialist and then we say, it's your choice'* P4
- *'I feel like in shared decision making there is a potential to confuse the patient with so much information and jargon that actually they leave not knowing. They're even more confused about what to do. They've been told about five to six different options, the advantages, disadvantages, they don't know the research, they don't know what's best for them and so I think a lot of them then make a poor choice or no choice at all because they're so confused'* P1.

Most participants felt they were practicing SDM regularly. However, conflicting statements suggested that the SDM model was being confused with informed consent:

- *'I give them all the pros and cons for each treatment option and [patients] have to make that decision and so eventually we do get to a point where they do make their decision by themselves and you know, with all the information I have given them'* P2
- *'Then leaving the decision with them is brilliant because then you've done your bit of actually informing them of everything, it's their decision now to either booking in the treatment or not'* P5.

All participants felt that the final decision always lay with the patient:

- *'Definitely the patient. It's always the patient. You can help them make that decision and you can make a recommendation but it's always the patient's decision at the end'* P9.

### Perceptions of regulatory bodies

Every participant expressed a degree of fear of being blamed as a reason to avoid taking responsibility for decisions. This fear of being blamed was directly linked to a fear of being sued which altered how options were communicated:

- *'I suppose maybe it's from a deep-rooted worry about being sued or having an issue or being in trouble with somebody or maybe them blaming me saying "well you told me to do this". I don't want the blame to be on me if something goes wrong as such, I want them to have made the informed decision themselv*es' P1
- *'Most people I feel I would push for them to have the simpler, more predictable option because it's not just their risk, it's your risk'* P8.

It appeared from the data that provision of all options/risks/benefits was perceived to be a medico-legal requirement, rather than what was going to facilitate decision making with the patient:

- *'Making sure patients are more aware of what you're doing so that it doesn't come back on you and be like, "you didn't tell me what you were doing initially". You don't want to get a complaint essentially so I think basically that is the driving force of it'* P7.

There was also acknowledgement from some participants that there was a conflict between different supporting bodies which made implementation of guidelines difficult:

- *'The fact is our regulatory body is not very clear about what it expects from us nor is the NHS and I think people are finding it difficult to have a proper conversation with their patients because of the unclear nature of our regulatory bodies'* P9.

### Dental care structure

Almost every participant commented on a lack of time as being a hindrance to delivery of effective communication:

- *'We've got 15 minutes for the check-up, by the time you get to talking about the different treatment options, once you've gone through the investigations, it's difficult to then discuss every single treatment option, the pros and cons, the costs and everything involved […] and then also enough time for them to ask all the questions that they may have. So it is very difficult with the time constraints, especially in NHS'* P2
- *'We're trying to keep everyone happy and I think that's a huge challenge because you can't run late and then you're constantly apologising for that or […] yeah I just find time pressure's more than anything because it affects your conversation, you'd love to explain a crown or root canal treatment in so much more detail than you can do but you've also got the pressure of knowing that five people are waiting in the waiting room and you know, you can't get enough information across quick enough to be [sic] to do it whereas if you've got more time, you can show diagrams, you can […] really get to the depth of things and patients make better clinical choices because of that'* P5.

There was generally an unfavourable view toward delivery of NHS care, often being the source of reasoning behind less effective communication:

- *'To meet targets, [dentists] tend to go down the paternalistic view of, on the NHS, trying to have less of the conversation, more, this is what you're having and go away and come back and we'll do it and it is very paternalistic, the old NHS system I think'* P9.

## Discussion

The participants of this study acknowledged SDM as a collaborative approach to decision making; however, when asked how their discussions with patients usually unfolded, what was described by most participants was a list of options given, together with risks and benefits and ultimately a decision which would be made by the patient. In fact, all participants believed that the patient ought to make the final decision. The process of informed consent is in line with the GDC,^17^ where there is an omission of actively identifying material risk, sharing responsibility for the decision and obtaining patient views and wishes*.* Therefore, this study suggests a possibility that the process of SDM is being misinterpreted as synonymous with informed consent. The literature also supports that this confusion between models of decision making can be due to a lack of a universally accepted definition for SDM.^12^

Most participants commented on the issue of conveying too much information to the patient which they believed could deter the patient from seeking treatment, owing to confusion or fear.^29^If the principle of SDM is to tailor information, this ought to filter out irrelevant information and thus streamline decision making. Wake and Wassif^30^ similarly discuss an 'interpretive approach' to discussion, where the patient's worries and needs are carefully evaluated. Given the recent Montgomery ruling,^31^ importance has been placed in determining what the 'reasonable person would want to know to make an informed choice'.^32^ The message is that by getting to know the patient, you can tailor information accordingly. Participants acknowledged that getting to know their patients over time facilitated discussions and made for easier communication. However, this process of getting to know your patients can take time and thus relies on rapport building. Therefore, SDM was more challenging when encountering patients for one-off appointments, such as emergency appointments, where little rapport was established. Conversely, more experienced participants found that decision making was more organic where they had been working in one practice for a long time, allowing such rapport building.

The delicate nature of the conversational 'dance'^3^ appears to rely on engagement from the practitioner and patient. Given the practitioner's role of having knowledge in a field, the onus is on the practitioner to have the skills to engage the patient in order to draw out information that places the patient in the context of their life.^33^ Results showed that this 'dance' has varying levels of complexity. One such factor was the perception of whether the patient wanted to engage in the appointment or not. Most participants appreciated that every patient is different and understood the importance of gauging how the patient wanted the discussion to unfold; so-called 'optional autonomy'.^34^ Where participants of this study found disengagement, a shift from SDM to giving blanket information occurred, which has also been reported.^35^

The literature supports the notion of patient choice, providing good communication has occurred.^11^ What is unclear is what constitutes good communication. By overstating risks in order to reduce expectation or 'nudge' the patient into selecting a predictable treatment option is a danger of impacting delivery of best possible care.^36^

Participants commented on their perception of the lack of, or conflicting, guidelines, which at times resulted in not knowing what was expected of them, in particular, between implementing some GDC standards^17^ with delivering NHS care. There was also a strong reaction toward a lack of support from such bodies, should patients complain.

This is consistent with the literature^11^^,^^37^ about the need for more guidance on what standards mean for patients, clinicians and others involved in delivering the service and how they can be supported to implement the desired aims. This confusion appears to result in a practice that is interpretative and subjective to the dentists' understanding of concepts/terms, with the main aim being to avoid potential complaints rather than support the patient who is at the centre of care.

Participants viewed the current NHS setting, particularly the GDS contract, as a barrier for delivering effective SDM. Consistent with the literature,^18^^,^^38^ participants reported that SDM was seen as time consuming, which made it less cost-effective in terms of what the participant was paid under the current contract, therefore becoming a barrier. Most participants who worked in private practices felt that they were compensated financially for their time and thus felt that SDM could be delivered without any (at least, financial) pressure.

There was an awareness that, while several patients were in the waiting room, there was a feeling of pressure which resulted in hurried conversations or perhaps more paternalistic conversations with the patient in surgery.^39^^,^^40^ What is unclear is whether this stress affects communication with patients and more research in this area is required.

Participants found that through dedicated time and seeing the patient regularly, trust and rapport were firmly established, permitting easier discussions. This is consistent with the bioethics literature describing trust and its relationship to enhanced autonomy and better decision making.^4^^,^^41^ Most participants supported patients who wanted more information and a desire for autonomy but did not relate the motivation to be from a source of mistrust. In fact, participants viewed a desire to be more involved as an opportunity to open up discussions and obtain an outcome in which the patient was happy and therefore reduced risk of blame. All participants supported patient autonomy. Their motivations for this support, however, were somewhat split. Some participants felt that the patient had an innate right to select treatment. Others felt a relief of legal burden by passing control to the patient. The outcome of both motivations was, nonetheless, the same; to reduce complaints.

Nearly every participant expressed an aversion to being responsible for solely deciding for their patients and if a decision could not be reached, most said they would advise their patients to seek a second opinion elsewhere. This is consistent with research done by Scambler *et al.*, where participants encouraged patients who were making the 'wrong' choice to comply or seek care elsewhere.^8^ This aversion to take on responsibility appears to arise from a fear of being blamed if something goes wrong during a course of treatment. Interestingly, however, when asked how participants would want their own care to be directed, most participants felt that paternalism still had a place in decision making. They acknowledged that they would want someone who knew their 'trade' to direct decision making or make the decision for them.

The purpose of promoting autonomy ought to be to empower the patient to take control of their own healthcare, which in turn, ought to result in better compliance and health outcomes.^42^ The purpose of SDM ought to be to promote autonomy between both dentist and patient so that evidence-based dentistry and patient lifestyle can become symbiotic. A by-product of SDM then ought to be a reduction in dentist and patient dissatisfaction because treatment that both parties have selected satisfy optimal delivery of care from the perspective of both dentist and patient. Participants of this study commented on feeling frustrated at providing no treatment on a tooth that clearly needed treatment. There was a sense that participants were obligated to promote autonomy at the expense of what they may have judged as best treatment.

The idea of promoting autonomy through fear of litigation appears to be enabling an ethos of defensive practice which may stifle the progress of dentistry in the UK. Ubel^43^ discusses in depth that when decisions become difficult, 'it is only natural for patients in such cases to look to their physicians for advice' and that this should be endorsed.

## Conclusion

Participants in this study supported SDM in principle; however, although they endorsed patient autonomy, there were connotations of defensive practice taking place which may be inhibiting true SDM. This defensive practice may be occurring due to a lack of understanding of SDM. Further, the communication process being delivered has a focus on delivery of information, for which there is evidence that it defeats the purpose of effective treatment selection and could potentially lead to poor decision making.

The results identified here need to be considered as an insightful snapshot rather than a representation of all dental practitioners, as more consideration and research are given to achieving a true patient-centered care approach, rather than a representation of all dental practitioners. The study demonstrates that SDM is misunderstood among participants as they focused on informed consent where SDM should be used, in order to tease out the best possible care for the individual patient. Several barriers were identified which inhibit SDM from taking place, including lack of time, with some tension between what the GDC expects of clinicians and what could actually be accomplished in the NHS setting according to participants. More education and support in relation to SDM, what it means and how it can enhance patient care is needed. This could be facilitated by using decision aids^44^^,^^45^ for both patients and practitioners to enhance the communication process. Imbedding patient-centred care and the concept of SDM throughout the curriculum, as well as continuing professional development, rather than stand-alone communication skills sessions, may serve to enhance clinicians' comprehension and implementation of SDM, bringing dental care more in line with wider health care. More research is needed to further explore factors influencing clinicians' understanding of SDM and how it interlinks with delivery of care.
